# The Influence of Depression, Positive Health Behaviors, and Weight Status on Glycated Hemoglobin: A Sequential Mediation Analysis of the INDEPENDENT Trial

**DOI:** 10.1007/s11606-025-09810-1

**Published:** 2025-08-13

**Authors:** Zach W. Cooper, Leslie C. M. Johnson, Shivani A. Patel, Sosale Ramachandra Aravind, Nikhil Tandon, Ranjit Mohan Anjana, Subramani Poongothai, Gumpeny R. Sridhar, Viswanathan Mohan, Lydia Chwastiak, Mohammed K. Ali

**Affiliations:** 1 University of Georgia School of Social Work, Athens, GA USA; 2 Emory University, Department of Family and Preventive Medicine, School of Medicine, Atlanta, GA USA; 3 Emory Global Diabetes Research Center, Woodruff Health Sciences Center and Emory University, Atlanta, GA USA; 4 Diacon Hospital, Bangalore, India; 5 Department of Endocrinology, All India Institute of Medical Sciences, New Delhi, India; 6 Madras Diabetes Research Foundation [ICMR- Collaborating Centre of Excellence] & Dr. Mohan’s Diabetes Specialties Centre [IDF Centre of Excellence in Diabetes Care], Chennai, Tamil Nadu India; 7 Endocrine and Diabetes Centre, Visakhapatnam, India; 8 Department of Psychiatry and Behavioral Sciences, University of Washington School of Medicine, Seattle, WA USA; 9 Hubert Department of Global Health, Emory University, Atlanta, GA USA

## Abstract

**Background:**

Collaborative care models (CoCM) improve both depression and cardiometabolic outcomes, but the mechanisms driving these effects remain unclear.

**Objective:**

To determine whether changes in depressive symptoms, positive health behaviors (PHBs), body mass index (BMI), and waist circumference mediate the effect of an adapted CoCM on glycemic control among adults with cardiometabolic risk in India.

**Design:**

Longitudinal structural equation modeling of data from the INtegrating DEPrEssioN and Diabetes TreatmENT (INDEPENDENT) Randomized Control Trial.

**Participants:**

Adults in India with type 2 diabetes or elevated cardiometabolic risk factors and clinically significant depressive symptoms.

**Interventions:**

An adapted CoCM integrating behavioral activation (BA) within diabetes care, delivered by a multidisciplinary team to enhance engagement in self-care activities.

**Main Measures:**

Intervention exposure was the independent variable; mediators were depressive symptoms (Symptom Checklist-20), PHBs (Summary of Diabetes Self-Care Activities), BMI, and waist circumference; the primary outcome was hemoglobin A1c (HbA1c). Confirmatory factor analyses ensured measurement validity, with second-order factors capturing depressive symptom domains (anhedonia, somatization, internalizing symptoms, restlessness).

**Key Results:**

The intervention significantly reduced depressive symptoms from 0–6 months (β = –0.22, *p* < 0.01) and 6–12 months (β = –0.34, *p* < 0.01) and increased PHBs from 0–6 months (β = 0.15, *p* < 0.05) and 6–12 months (β = 0.11, *p* < 0.05). Higher depressive symptoms predicted lower PHBs at 6 months (β = –0.26, *p* < 0.01) and 12 months (β = –0.25, *p* < 0.01). PHBs mediated reductions in BMI (β = –0.08 to –0.09, *p* < 0.01) and waist circumference, which in turn mediated improvements in HbA1c.

**Conclusions:**

An adapted CoCM improved glycemic control and cardiometabolic risk by alleviating depressive symptoms, enhancing PHBs, and reducing BMI and waist circumference. Integrating BA within CoCM may be a key mechanism for optimizing outcomes in integrated cardiometabolic care.

**Supplementary Information:**

The online version contains supplementary material available at 10.1007/s11606-025-09810-1.

## BACKGROUND

Diabetes is a highly prevalent chronic medical condition, affecting over 537 million individuals globally and leading to almost a trillion dollars in annual healthcare expenditures^1^. Type 2 diabetes (T2D) accounts for approximately 95% of diabetes worldwide and up to 65% of people with T2D experience depressive symptoms^1–3^. In India alone, over 200 million people are reported to have diabetes^4^. However, over 50% of individuals with T2D indicate that they do not receive care for their depressive symptoms as part of their routine diabetes care^3^. Untreated depression among patients with diabetes is associated with difficulties regarding self-management leading to complications in diabetes care^5^.

Integrated care (IC) models address physical and mental health needs by embedding behavioral health professionals within medical teams^6^. IC models range in their levels of integration from increasing collaboration between primary and specialty mental health settings, to co-locating mental health professionals in the same building with different workflows, to fully integrating behavioral health professionals onto medical teams with shared workflows and treatment plans^6,7^. Behavioral health professionals working within IC models adapt evidence-based interventions such as Solution-Focused Brief Therapy^8^, Behavioral Activation (BA)^9,10^, Acceptance and Commitment Therapy^11^, and Cognitive Behavioral Therapy^12^ to align with medical workflows (shorter and less frequent sessions).

IC models have shown efficacy in improving both depressive symptoms and glycemic control in patients with diabetes and comorbid depression^9,10,13,14^. However, most clinical trials evaluate the overall outcomes of IC models without investigating the underlying change mechanisms—i.e., the behavioral processes by which interventions improve health. While the former approach confirms the effectiveness of IC models, it overlooks the specific components driving those outcomes. Understanding these components is important due to the variation in the types of IC models and behavioral health interventions utilized within these models. Further, current research typically treats depression as a unidimensional construct, missing the opportunity to explore how IC models impact different symptom profiles (e.g., anhedonia [loss of interest or pleasure], somatic [physical symptoms like fatigue], and cognitive symptoms [negative thoughts and concentration difficulties]). Given this heterogeneity in depressive presentations, examining how IC models influence specific symptom domains is crucial.

Existing studies have adapted IC models for in the United States^15,16^, Spain^17,18^, Nepal^19^, and the United Kingdom^20,21^, with adaptations for low and middle-income countries limited to India^10,22^. However, these studies rarely examine the underlying mechanisms that demonstrate how IC models improve patient health outcomes^14^. Further, no identified study has examined change mechanisms explaining the pathways through which IC models work for people in these settings, despite their unique treatment needs.

Our study conducts a longitudinal mediation analysis of the Integrating Depression and Diabetes Treatment (INDEPENDENT) Randomized Clinical Trial^10^. The INDEPENDENT adapted a Collaborative Care Model (CoCM) for patients in India, demonstrating improvements in both cardiometabolic health and depressive symptoms^10^. Using data from this trial, we investigate the mechanisms of change driving cardiometabolic outcomes. Grounded in prior research that links diabetes outcomes to health behaviors^23^, depression^24^, body mass index [BMI], and waist circumference^25^, this study examines how changes in depressive symptoms, health behaviors, BMI, and waist circumference impact hemoglobin A1c (HbA1c). By exploring a sequential causal pathway, we aim to clarify how the CoCM intervention affects HbA1c through reductions in depressive symptoms, promotion of positive health behaviors, and reductions in weight.

## METHODS

**Figure 1:**
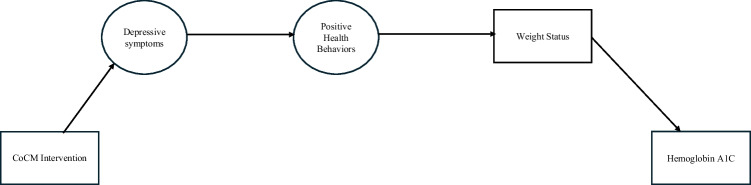
Conceptual Mediation Model. Final mediation path model analyzing the direct and indirect effects of the collaborative care model (CoCM) intervention. All circles represent a latent variable included in our path model. All boxes represent a manifest variable in our model. Single-headed arrows represent a causal pathway.

This study is a secondary analysis of the INDEPENDENT study, a multicenter, open-label, pragmatic trial with patient-level randomization conducted across four urban diabetes clinics in India^26^. Using a structural equation model (SEM), we examine how the CoCM intervention from the INDEPENDENT Trial influences diabetes outcomes. Specifically, we investigate the pathways linking the intervention to diabetes outcomes through three mediating constructs: 1) depressive symptoms, 2) positive health behaviors, 3) BMI, and 4) waist circumference, Fig. 1 presents the conceptual model. Trial data were collected at baseline (0 months), 6 months, 12 months, 24 months, and 36 months, however, this analysis focuses on the active intervention period (0–12 months) to identify key mechanisms of change driving the CoCM’s effects.

### The INDEPENDENT Trial

Within the INDEPENDENT trial, 404 patients were randomized at the patient level to either receive a collaborative care intervention (n = 196) or treatment as usual (n = 208). Of the 404 patients, 378 completed follow-ups and were included in the final analysis. See Supplemental File 1for the trial CONSORT diagram^10^. Inclusion criteria indicated that participants were 1) 35 or older, 2) had one or more poorly controlled cardiometabolic indicators (HbA1c ≥ 8%, SBP ≥ 140 mm Hg, or LDL-c ≥ 130 mg/dl), and 3) had clinically significant depressive symptoms (a Patient Health Questionnaire-9 score of ≥ 10). Patients were excluded from the trial if they had a history of mania, psychosis, or dementia, or were currently receiving treatment by a psychiatrist. A detailed description of the main trial outcomes have been previously published^26^.

### Intervention Description

The INDEPENDENT trial utilized a CoCM, a specific type of IC model that fully integrates a behavioral health care coordinator within the medical team^10,27^. The intervention comprised multiple components, including enhanced care coordination, measurement-based care, behavioral activation, motivational interviewing, patient education, clinical decision-making support, specialty consultation, and expert case review meetings^10^. In terms of delivery, a non-physician care coordinator conducted patient visits every 2–4 weeks, delivered behavioral interventions, monitored patient progress, and communicated this information to the patients’ primary diabetes clinician, a consulting psychiatrist, and a senior endocrinologist^28,29^.

Behavioral activation was the primary behavioral intervention employed to support diabetes self-management behavior. Behavioral activation is a brief, evidence-based approach that helps individuals with depression and chronic illness develop skills to re-engage in personally meaningful and valued activities^30,31^. Sessions typically involve four core components: 1) identifying individual values, 2) translating those values into specific, meaningful activities, 3) linking these activities to health-promoting behaviors, and 4) setting actionable goals to initiate those behaviors^31^. BA sessions are shorter in duration (approximately 20–30 min), less frequent than traditional psychotherapy, and can be effective when delivered in a single session. Prior qualitative analyses have shown that participants found the INDEPENDENT care model empowering and engaging, reporting increased activation and improved engagement in their diabetes care^32^.

## MEASURES

### Treatment Assignment

Treatment Assignment is a dichotomous outcome, with those who were exposed to treatment as usual labeled as “0” and those who were in the intervention group labeled as “1.” Since the assignment did not change throughout the study, it only serves as a baseline predictor.

### Hemoglobin A1C

HbA1c provides an average of the patient’s blood glucose levels over the past 3 months and is commonly used as a clinical measure of diabetes severity^33^. HbA1c is a continuous variable with typical ranges from 6.5%−14% for those who have a diagnosis of diabetes^34^. Consequently, we included HbA1c as the primary outcome measure for diabetes health.

### Mediators

Three key constructs serve as mediators in our model: depressive symptoms, positive health behaviors, and weight (as indicated by BMI and waist circumference). These mediators are ordered sequentially, grounded in the theory that the intervention first reduces depressive symptoms, which subsequently leads to improvements in health behaviors and a reduction in weight status, see Fig. 1.

### Depressive symptoms (M1)

We utilized the Symptom Checklist-20 (SCL-20) to analyze changes in depressive symptoms^35^. The SCL-20 utilizes a 5-point Likert scale for each question ranging from “0 = Not at all” to “4 = Extremely.” In previous studies, the SCL-20 has demonstrated acceptable validity and sensitivity for measuring depressive symptoms^35^. Existing research has utilized second-order constructs (e.g. somatization; anhedonia) to improve construct reliability and validity and increase precision for measuring depressive symptoms with the Patient Health Questionnaire-9^36^and the Beck Depression Inventory^37^. We utilized these studies to inform the creation of second-order constructs for the SCL-20 in our sample. Including second-order constructs improved the reliability and validity of the measure (χ2(138, N = 371) = 273.51; RMSEA = 0.051(0.042-0.060); SRMR = 0.058; CFI = 0.939) and enabled us to analyze intervention and mediating effects for different symptom profiles of depression, see Supplemental File 2. The SCL-20 also demonstrated sufficient internal reliability with a baseline Cronbach alpha of 0.82 which was maintained when repeated at 12 months. Last, we acquired a test–retest coefficient which demonstrated good test–retest reliability (r = 0.67).

### Positive Health Behaviors (M2)

The Summary of Diabetes Self-Care Activities (SDSCA) scale was used to measure positive health behaviors^38^. In previous studies, the SDSCA has demonstrated significant construct validity and inter-item reliability^38^. The scale includes constructs of Positive Health Behaviors such as healthy eating (following an eating plan, eating fruits and vegetables), exercise, and treatment adherence (checking blood sugars, and foot care). Each of these measures uses a 7-point scale and asks, “How many days in the past week did you engage in ____ behavior,” with a separate item for each behavior. Within our sample, the SDSCA demonstrated acceptable construct validity and reliability, see Table 1. In addition, the measure demonstrated good internal reliability with an alpha of 0.82 which was maintained when repeated. Last, the measure demonstrated good test–retest reliability (r = 0.62).

**Table 1:** Model Fit Indices

Model	df	χ2	CFI	TFI	SRMR	RMSEA	Factor Loading Range
**Confirmatory Factor Analysis**							
Depression as a single factor	190	396.78	.893	.871	.068	.064	-.05 to.63
Depression with second-order constructs	138	273.51	.939	.924	.058	.051	.17 to.88
Positive Health Behaviors	279	823.49	.945	.931	.075	.073	.15 to.82
Full Model with second-order constructs	301	273.51	.910	.900	.067	.053	.16 to.88
Longitudinal with second-order constructs	2985	5111.74	.901	.899	.070	.042	.18 to.91
**Structural Equation Model**							
Complex Mediation	3309	5930.71	.871	.867	.079	.045	.18 to.95
Simplified Model	3309	5882.76	.901	.899	.075	.042	.19 to.95

### Weight Status: Body Mass Index or Waist Circumference (M3)

Measures of BMI and waist circumference are known cardiometabolic risk factors, with higher measures being associated with a higher risk of developing diabetes or cardiovascular disease^39^. Consequently, we performed an analysis that examined BMI as a primary indicator of weight status and another analysis that utilized waist circumference as the indicator of weight status. The results from both analyses are presented in the results section for comparison.

## ANALYSIS

Univariate statistics were conducted for each study variable to confirm no significant violations of normality. Bivariate analyses were then performed to ensure no significant differences in clinical and demographic characteristics between the treatment and control groups.

We conducted confirmatory factor analyses (CFA) for each latent variable in the final mediation model—depression and positive health behaviors. We also developed second-order latent constructs for depression, including internalized depression, anhedonia, somatic symptoms, and restlessness. This allowed us to distinguish between different facets of depression, which may respond differently to treatment and have varying impacts on health behaviors and overall disease management. A longitudinal CFA was then conducted to evaluate the consistency of our latent constructs (depressive symptoms and positive health behaviors) across time points. Measurement invariance was tested at 0, 6, and 12 months.

Once reliability and validity were confirmed, we tested the mediating effects of depressive symptoms, health behaviors, BMI, and waist circumference on diabetes outcomes using a complex longitudinal mediation model, including several covariates. We then compared this with a simplified, more parsimonious model that retained only the key regression paths essential to our hypothesis, eliminating unnecessary covariates. The simplified model demonstrated superior fit, and its results are presented throughout the manuscript. All parameter estimates are standardized to account for differences in scale among the latent constructs. Missing data were handled using the Full Information Maximum Likelihood (FIML) method^40^. All analyses were performed using the Lavaan package in R version 4.2.3^41^.

## RESULTS

### Confirmatory Factor Analyses (CFAs)

When comparing the single latent construct model to the second-order model, the second-order model exhibited a significantly better fit (χ^2^ (52, N = 371) = 123.27, *p* < 0.001). A CFA of the positive health behaviors scale demonstrated strong construct validity and reliability (see Table 1). Subsequently, we tested a combined CFA model incorporating both depressive symptoms and positive health behaviors, which yielded acceptable reliability and validity (χ^2^(301, N = 371) = 273.51; RMSEA = 0.053, 90% CI [0.047, 0.059]; CFI = 0.910), see Fig. 1 and Table 1.

Finally, we extended the analysis to a longitudinal CFA, including data collected at 0, 6, and 12 months, to evaluate the stability of these constructs over time. The longitudinal CFA demonstrated adequate fit (χ^2^(2985, N = 371) = 5111.74; RMSEA = 0.042, 90% CI [0.040, 0.046]; CFI = 0.90), supporting the consistent measurement of depressive symptoms and health behaviors throughout the intervention period, see Supplemental File 3.

### Longitudinal Mediation Model

#### Intervention Effects on Mediators and Dependent Variables

The CoCM intervention had a significant direct effect on HbA1c during the initial 0–6-month period (β = −0.27, *p* < 0.01). However, this effect diminished between 6–12 months, and the change in HbA1c was no longer significant at 12 months (β = −0.04, *p* = 0.64). We then incorporated mediators (depressive symptoms, health behaviors, BMI, and waist circumference) and covariates into a more complex model. When including the mediators, the direct effect of the intervention on HbA1c was reduced (β = −0.07, *p* < 0.07), reflecting that changes in depressive symptoms, health behaviors, BMI, and waist circumference accounted for part of the treatment effect. In this model, the CoCM intervention had significant effects on depressive symptoms, with reductions from 0–6 months (β = −0.23, *p* < 0.01) and sustained improvements from 6–12 months (β = −0.34, *p* < 0.01), indicating that those in the intervention group experienced a notable decrease in depression scores. Similarly, the intervention significantly improved positive health behaviors during both periods, with effects from 0–6 months (β = 0.15, *p* < 0.05) and 6–12 months (β = 0.11, *p* < 0.05), suggesting an increase in adherence to healthy behaviors like diet and exercise over time. There were no significant intervention effects on BMI or waist circumference.

#### Effects on Second-order Constructs of Depressive Symptoms

We also examined intervention effects on specific depressive symptom profiles, using second-order constructs (anhedonia, restless symptoms, somatic symptoms, and internalized depression). The CoCM significantly reduced anhedonia from 0–6 months (β = −0.12, *p* < 0.05) and from 6–12 months (β = −0.22, *p* < 0.01). However, symptoms of restlessness, somatic complaints, and internalizing cognitions were more resistant to change. We performed a sensitivity analysis examining weight status as a latent factor that yielded no significant effects, See Supplemental Files 4 and 5.

#### Longitudinal Mediation Effects

In analyzing the longitudinal mediation effects, we found significant relationships between depressive symptoms and health behaviors over time. Depressive symptoms had a negative effect on health behaviors from baseline to 6 months (β = −0.28, *p* < 0.01) and from 6 to 12 months (β = −0.27, *p* < 0.01), indicating that reductions in depressive symptoms significantly improved positive health behaviors. Health behaviors, in turn, mediated the relationship between depressive symptoms and weight. Specifically, there was a significant effect of health behaviors on BMI (β = −0.08, *p* < 0.05) and waist circumference (β = −0.07, *p* < 0.05) from both 0 to 6 months and 6 to 12 months.

When examining second-order constructs of depressive symptoms, we found that restless symptoms (β = −0.33, *p* < 0.01) and somatic symptoms (β = −0.33, *p* < 0.01) significantly mediated the relationship between depression and health behaviors during the 0–6-month period, with similar effects observed from 6–12 months. Other depressive symptom profiles did not show significant mediation effects. The waist circumference model showed similar directional and magnitude effects for secondary depressive factors, see Table 2. We also conducted a sensitivity analysis with weight status modeled as a latent variable, which produced comparable mediation effects, see Supplemental File 4. Lastly, covariates were included in the complex model for both BMI and waist circumference, with results available in Supplemental File 6.

**Table 2:** Results from Simplified Model

**BMI Model**						
**Path Descriptions**	**Path**	**Depression**	**Anhedonia**	**Restless**	**Somatic**	**Int. Dep**
**Intervention Paths**						
Treatment condition predicts depressive symptoms (a path)	a1	−0.02	0.02	0.01	−0.03	0.04
	a2	−0.22**	−0.12*	0.05	−0.04	0.03
	a3	−0.34**	−0.22**	−0.01	0.01	0.02
Treatment condition predicts positive health behaviors (b path)	b1	0.02	0.02	0.02	0.02	0.02
	b2	0.15*	0.15*	0.15*	0.15*	0.15*
	b3	0.11*	0.10	0.10	0.10	0.10
Treatment condition predicts BMI (d path)	d1	−0.01	−0.01	−0.01	−0.01	−0.01
	d2	−0.01	−0.01	−0.01	−0.01	−0.01
	d3	0.02	0.02	0.02	0.02	0.02
Treatment condition predicts A1C (c’ path)	c’1	0.08	0.09	0.08	0.08	0.08
	c’2	−0.07	−0.06	−0.07	−0.06	−0.07
	c’3	−0.10	−0.08	−0.09	−0.10	−0.10
**Mediation Paths**						
Depression mediates Health behaviors (h path)	h1	−0.26**	−0.05	−0.33**	−0.09	−0.03
	h2	−0.25**	−0.05	−0.36**	−0.07	−0.02
Health Behaviors mediate BMI (i path)	i1	−0.08*	−0.07*	−0.07*	−0.07*	−0.07*
	i2	−0.09*	−0.08*	−0.08*	−0.07*	−0.08*
**Waist Circumference Model**						
Treatment condition predicts depressive symptoms (a path)	a1	−0.03	0.02	0.01	−0.04	0.04
	a2	−0.22**	−0.12*	0.05	−0.04	0.03
	a3	−0.34**	−0.22**	−0.01	0.01	0.02
Treatment condition predicts positive health behaviors (b path)	b1	0.02	0.02	0.02	0.02	0.02
	b2	0.15*	0.15*	0.15*	0.15*	0.15*
	b3	0.11*	0.11*	0.10	0.10	0.10
Treatment condition predicts WC (d path)	d1	0.05	0.05	0.05	0.03	0.05
	d2	−0.01	−0.01	−0.01	−0.02	−0.01
	d3	−0.02	−0.02	−0.02	−0.02	−0.02
Treatment condition predicts A1C (c’ path)	c’1	0.09	0.09	0.08	0.08	0.08
	c’2	−0.07	−0.07	−0.12*	−0.12*	−0.13*
	c’3	−0.10	−0.10	−0.09	−0.10	−0.10
**Mediation Paths**						
Depression mediates Health behaviors (h path)	h1	−0.28**	−0.06	−0.33**	−0.19*	−0.04
	h2	−0.27**	−0.06	−0.35**	−0.01	−0.03
Health Behaviors mediate WC (i path)	i1	−0.08*	−0.07*	−0.07*	−0.02	−0.07*
	i2	−0.08*	−0.07*	−0.07*	−0.02	−0.07*

*p <.05,**p <.01

We compared the simplified and complex model and found the simplified model had a better overall fit (χ2(3836, N = 371) = 5882.76; RMSEA = 0.044(0.042-0.046); CFI = 0.91). As a result, we incorporated the effects from the simplified and more parsimonious model into Table 2.

## DISCUSSION

Our longitudinal mediation analysis revealed the significant mediating roles of depressive symptoms, health behaviors, and weight (BMI or waist circumference) of the Collaborative Care Model (CoCM). Notably, the CoCM intervention reduced depressive symptoms, which in turn promoted positive health behaviors. These health behaviors facilitated reductions in both BMI and waist circumference, which together accounted for a significant portion of the HbA1c reduction achieved by the CoCM intervention. Additionally, by incorporating second-order constructs of depressive symptoms, we enhanced the precision and reliability of depression measurement, allowing for a more nuanced analysis of symptom-specific effects. Our findings indicate that the CoCM was particularly effective in alleviating anhedonia, while restless symptoms had the strongest influence on improving health behaviors but were resistant to significant treatment effects.

Several trials have demonstrated the efficacy of CoCMs for improving diabetes and depression outcomes^9,14,42^These studies, however, lack a comprehensive analysis of how treatment effects were achieved and which depressive symptoms are influential in determining treatment effects. Our findings from the INDEPENDENT study suggest that while the CoCM intervention was particularly effective in alleviating anhedonia; symptoms related to internalized cognitions, somatic complaints, and restlessness were more resistant to change. This indicates that additional therapeutic approaches may be necessary to fully address the diverse symptom profiles of depression in patients with diabetes. The INDEPENDENT trial utilized behavioral activation as the primary therapeutic intervention^10^, a practice consistent with other trials of CoCMs^9,14,42^. Since behavioral activation primarily targets anhedonia, clinicians should consider integrating complementary therapeutic approaches, such as Acceptance and Commitment Therapy or Mindfulness-Based Cognitive Behavioral Therapy, to address internalized, somatic, and restless symptoms. These therapies focus on cognitive processing and coping mechanisms, which may enhance treatment outcomes for patients who exhibit these less responsive symptoms^43–46^.

Our study also examined intervention effects on health behaviors and weight while analyzing the mediating effects on healthcare outcomes. Existing research has examined the effects of CoCM and other integrated care models on treatment adherence (e.g. taking medications, eating healthy)^47^. However, these studies typically evaluate medication adherence, eating behaviors, and other health behaviors as separate, manifest variables^48^. To address this gap, we examined positive health behaviors as a latent variable (e.g. healthy eating, exercise, and checking blood sugars) and evaluated it as a mediating construct. Our findings highlight the crucial role of depressive symptom reduction in improving health behaviors, such as healthy eating, exercise, and blood sugar monitoring, which in turn led to significant reductions in both BMI and waist circumference. These results highlight the necessity within clinical practice of adequately addressing depressive symptoms among patients with T2D, as improvements in depressive symptoms may indirectly drive better self-management behaviors and weight reduction. Moreover, the findings emphasize the importance of screening for and treating depression in patients with diabetes who face challenges in self-management, as depressive symptoms—particularly anhedonia—can impede adherence to critical health behaviors such as medication compliance, diet, and physical activity.

## LIMITATIONS

This study is not without limitations. The study sample size was powered for the main trial outcomes, leaving a smaller sample (n = 371) for mediation analysis. Though SEM power analyses indicate that it is feasible to perform complex structural models with this sample size, it is possible that some of the effects were not detected. We used bootstrapping procedures to address this limitation. In addition, positive health behaviors were examined as a single latent construct because we did not have enough variables to assess second-order constructs of health behaviors. Future studies may utilize scales with more items to evaluate positive health behaviors more precisely. Last, this study did not collect a full metabolic profile with biomarkers. Future studies may seek to collect biomarker data to create latent variables to model metabolic profiles.

## CONCLUSIONS

The CoCM adapted for patients in India had a significant treatment effect on depressive symptoms, with the greatest impact on reducing symptoms of anhedonia. By reducing depressive symptoms, the intervention also had a downstream effect on positive health behaviors and weight status. Depression and positive health behaviors had a strong partial mediating effect on healthcare outcomes, providing additional explanation of the active ingredients of the CoCM. CoCMs provide an effective method to increase access to mental health where resources may be scarce, with benefits for people with diabetes who need support reducing depressive symptoms and mobilizing health behaviors.

## Supplementary Information

Below is the link to the electronic supplementary material.

Supplementary file1 (PDF 172 KB)

Supplementary file2 (PDF 252 KB)

Supplementary file3 (PDF 100 KB)

Supplementary file4 (DOCX 21 KB)

Supplementary file5 (PDF 118 KB)

Supplementary file6 (DOCX 28 KB)

## Data Availability

Data is available upon reasonable request. Interested parties can contact Zach Cooper, the corresponding author, for further information.
