# Supplementary material for: The Influence of Depression, Positive Health Behaviors, and Weight Status on Glycated Hemoglobin: A Sequential Mediation Analysis of the INDEPENDENT Trial
Source: J Gen Intern Med. 2025 Aug 13;40(15):3715–22. doi: 10.1007/s11606-025-09810-1 (PMC12612419; doi:10.1007/s11606-025-09810-1)

Supplemental File 1: CONSORT diagram explaining the recruitment and randomization process for the INDEPENDENT clinical trial[28]

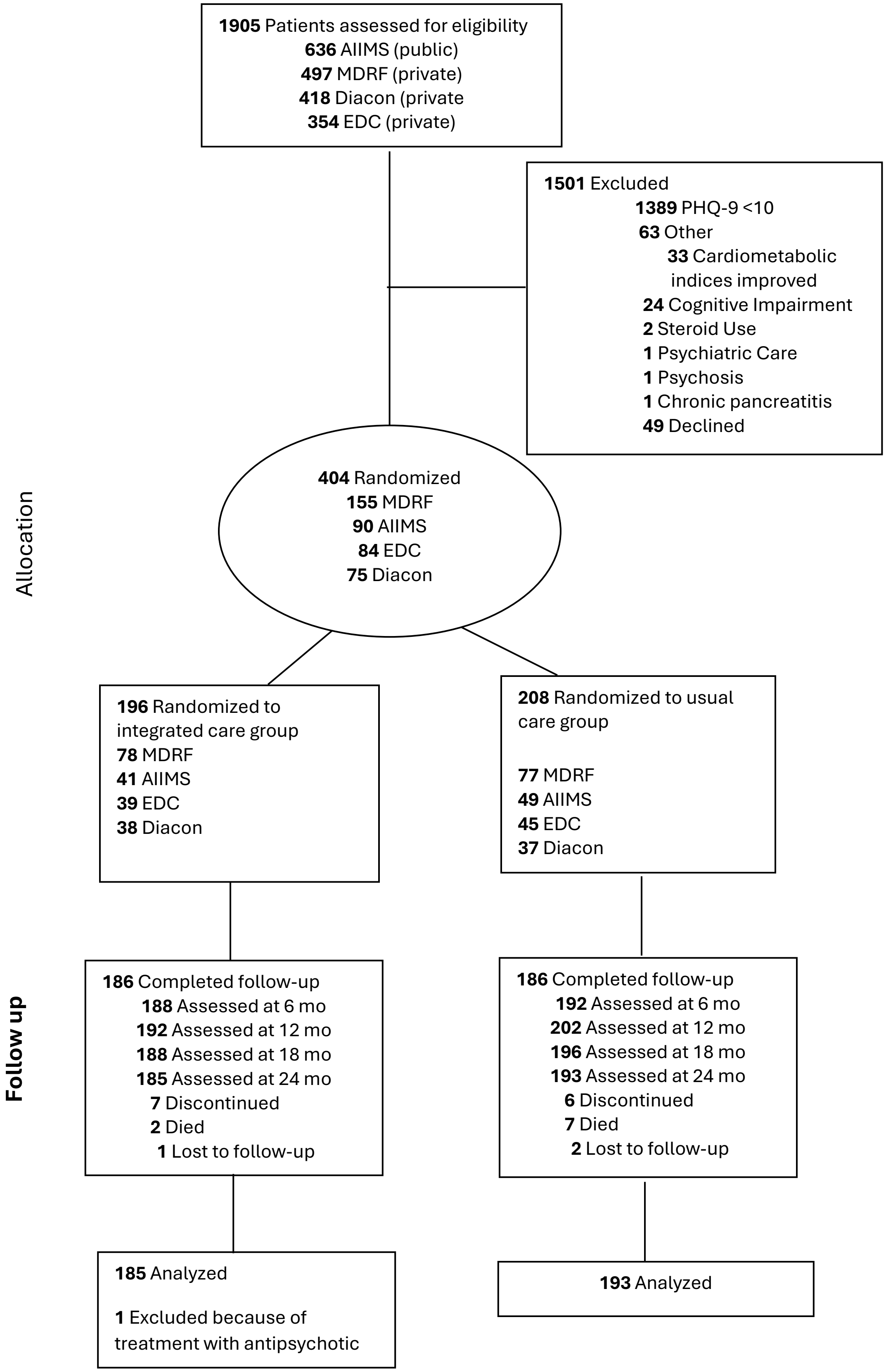

Supplement: Supplementary file 1 — Supplementary file1 (PDF 172 KB) [file 11606_2025_9810_MOESM1_ESM.pdf]
