# Supplementary material for: The Influence of Depression, Positive Health Behaviors, and Weight Status on Glycated Hemoglobin: A Sequential Mediation Analysis of the INDEPENDENT Trial
Source: J Gen Intern Med. 2025 Aug 13;40(15):3715–22. doi: 10.1007/s11606-025-09810-1 (PMC12612419; doi:10.1007/s11606-025-09810-1)

Supplemental File 2: Cross-sectional confirmatory factor analysis (CFA) testing the validity and factor structure of the second-order depression model.
-All Circles represent latent variables within our CFA
-All boxes represent a manifest variable
-Each single-headed arrow represents a factor loading
-Double-headed arrows featured below manifest variables represent residual variances

Model Fit: χ2(138, N=371) = 273.51; RMSEA = .051(.042-.060); SRMR = .061; CFI = .939; TFI = .924

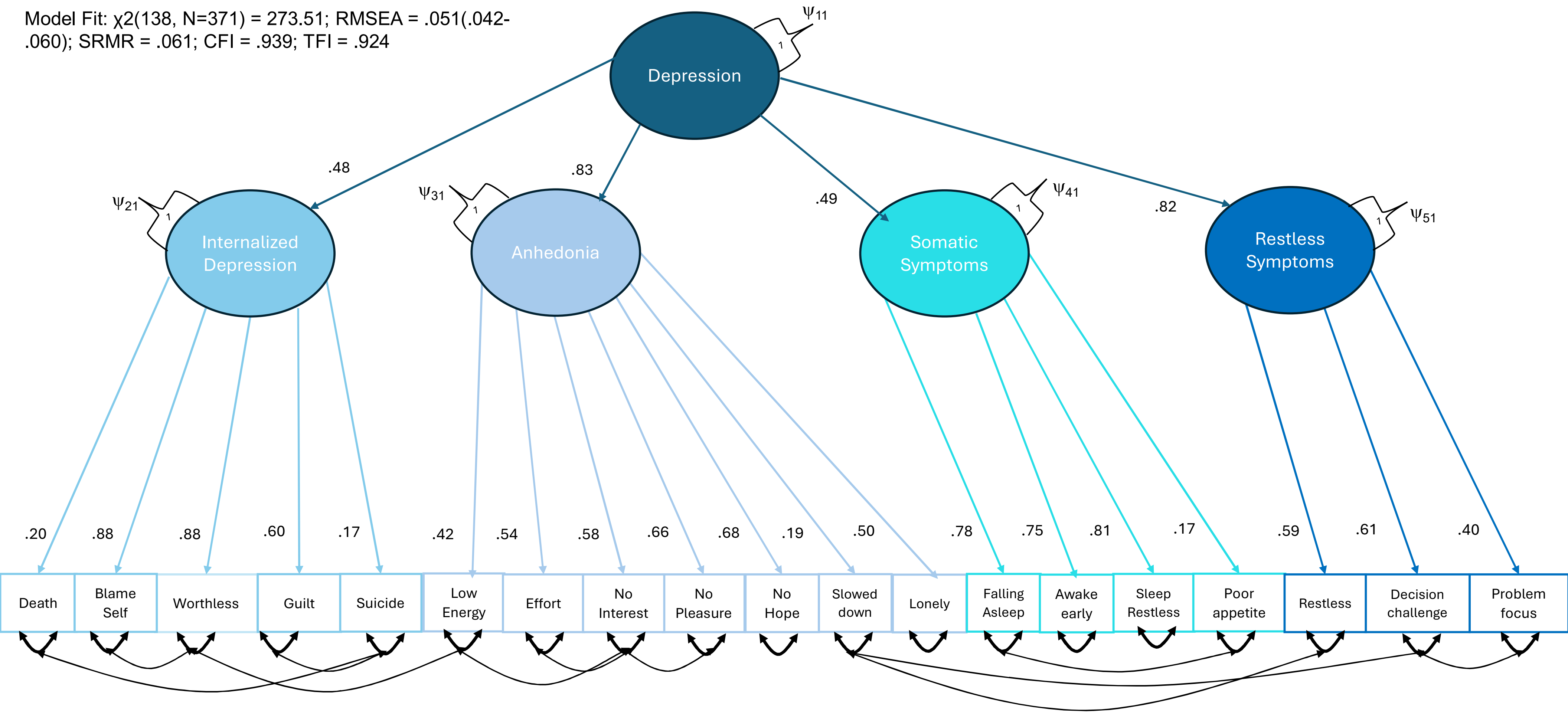

Supplement: Supplementary file 2 — Supplementary file2 (PDF 252 KB) [file 11606_2025_9810_MOESM2_ESM.pdf]
