# Supplementary material for: The Influence of Depression, Positive Health Behaviors, and Weight Status on Glycated Hemoglobin: A Sequential Mediation Analysis of the INDEPENDENT Trial
Source: J Gen Intern Med. 2025 Aug 13;40(15):3715–22. doi: 10.1007/s11606-025-09810-1 (PMC12612419; doi:10.1007/s11606-025-09810-1)
Supplement: Supplementary file 3 — Supplementary file3 (PDF 100 KB) [file 11606_2025_9810_MOESM3_ESM.pdf]

Supplement 3: Longitudinal Confirmatory Factor Analysis

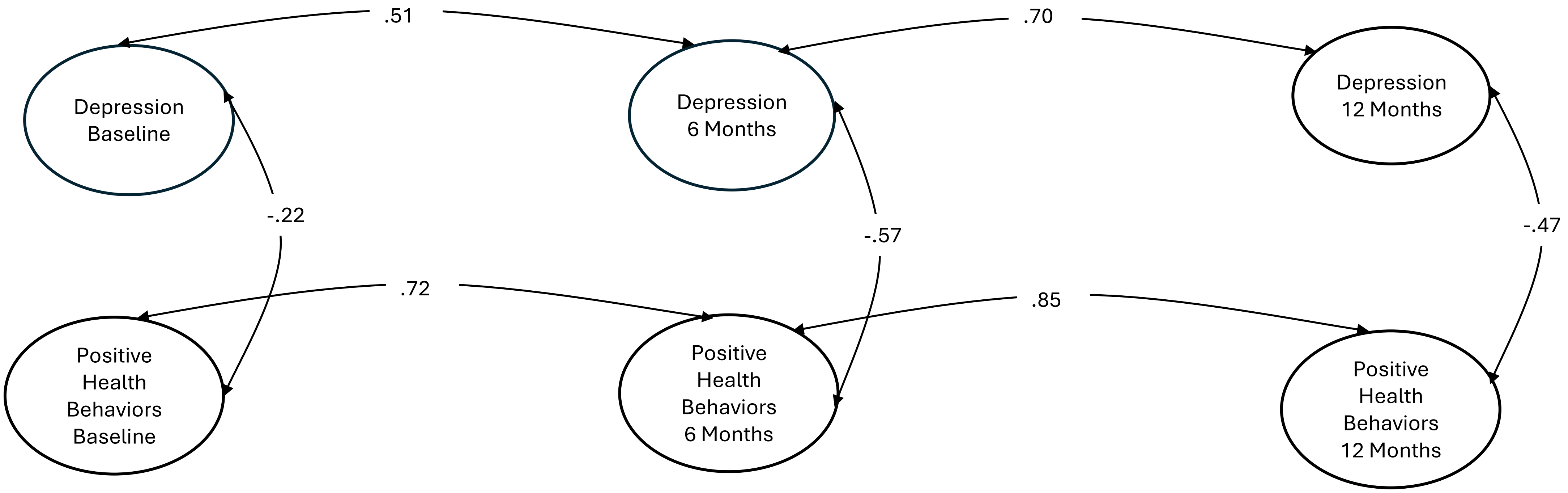

Supplemental File 3- Longitudinal Confirmatory Factor Analysis (CFA) examining the validity of depression and positive health behaviors through the trial while examining the relationship between these variables across the course of the study

-All circles represent a latent variable within our CFA model
-Double-headed arrows connecting two latent variables represent covariances
*All values are statistically significant
