# Supplementary material for: The Influence of Depression, Positive Health Behaviors, and Weight Status on Glycated Hemoglobin: A Sequential Mediation Analysis of the INDEPENDENT Trial
Source: J Gen Intern Med. 2025 Aug 13;40(15):3715–22. doi: 10.1007/s11606-025-09810-1 (PMC12612419; doi:10.1007/s11606-025-09810-1)
Supplement: Supplementary file 4 — Supplementary file4 (DOCX 21 KB) [file 11606_2025_9810_MOESM4_ESM.docx]

**Supplement 4: Sensitivity Analyses for Adiposity**

| **Model** | **Path** | **Depression** | **Anhedonia** | **Restless** | **Somatic** | **Int. Dep** |
| --- | --- | --- | --- | --- | --- | --- |
| **Intervention Paths** | | | | | | |
| Treatment condition predicts depressive symptoms (a path) | a1 | -0.07 | 0.02 | -0.02 | -0.04 | 0.02 |
|  | a2 | **-0.24**** | **-0.11*** | 0.01 | -0.03 | 0.03 |
|  | a3 | **-0.34**** | **0.23**** | -0.01 | 0.02 | 0.02 |
| Treatment condition predicts positive health behaviors (b path) | b1 | -0.02 | -0.01 | -0.01 | -0.02 | -0.01 |
|  | b2 | **0.15*** | **0.13*** | **0.14*** | **0.13*** | **0.13*** |
|  | b3 | **0.11*** | 0.05 | 0.06 | 0.05 | 0.05 |
| Treatment condition predicts adiposity (d path) | d1 | 0.06 | 0.04 | 0.04 | 0.04 | 0.04 |
|  | d2 | 0.05 | 0.04 | 0.04 | 0.04 | 0.04 |
|  | d3 | 0.05 | 0.05 | 0.05 | 0.05 | 0.05 |
| Treatment condition predicts A1C (c’ path) | c’1 | 0.09 | 0.09 | 0.09 | 0.08 | 0.08 |
|  | c’2 | **-0.11*** | **-0.14*** | **-0.12*** | **-0.12*** | **-0.12*** |
|  | c’3 | -0.09 | -0.08 | -0.10 | -0.10 | -0.10 |
| **Concurrent Paths** | | | | | | |
| Depression predicts A1C (e path) | e1 | 0.04 | 0.06 | 0.01 | 0.03 | 0.02 |
|  | e2 | **0.11*** | **0.12*** | 0.09 | 0.08 | 0.06 |
|  | e3 | 0.03 | 0.03 | 0.04 | 0.01 | 0.07 |
| Health Behaviors predict A1C (f path) | f1 | 0.07 | 0.08 | 0.10 | 0.07 | 0.07 |
|  | f2 | -0.02 | -0.02 | 0.01 | -0.03 | -0.06 |
|  | f3 | -0.08 | -0.01 | -0.08 | -0.02 | -0.01 |
| Adiposity Predicts A1C (g path) | g1 | -0.05 | -0.03 | -0.06 | -0.04 | -0.03 |
|  | g2 | -0.04 | -0.02 | -0.02 | -0.02 | -0.02 |
|  | g3 | 0.01 | 0.03 | 0.01 | 0.04 | 0.04 |
| **Cross-Lagged Paths** | | | | | | |
| Depression mediates Health behaviors (h path) | h1 | **-0.23**** | 0.08 | **-0.16**** | **-0.12*** | -0.03 |
|  | h2 | **-0.22**** | -0.05 | **-0.13**** | -0.09 | -0.05 |
| Health Behaviors mediate adiposity (i path) | i1 | **-0.09*** | **-0.09*** | **-0.09*** | **-0.09*** | **-0.09*** |
|  | i2 | **-0.10*** | **-0.10*** | **-0.10*** | **-0.10*** | **-0.10*** |
| **Autoregressive Paths** | | | | | | |
| Depression (j path) | j1 | **0.62**** | **0.62**** | **0.62**** | **0.62**** | **0.62**** |
|  | j2 | **0.74**** | **0.74**** | **0.74**** | **0.74**** | **0.74**** |
| Health Behaviors (k path) | k1 | **0.92**** | **0.92**** | **0.92**** | **0.92**** | **0.92**** |
|  | k2 | **0.98**** | **0.98**** | **0.98**** | **0.98**** | **0.98**** |
| Adiposity (l path) | l1 | **0.97**** | **0.97**** | **0.97**** | **0.97**** | **0.97**** |
|  | l2 | **0.98**** | **0.98**** | **0.98**** | **0.98**** | **0.98**** |
| **Supplemental File 4-** A results table summarizing the effect sizes for the sensitivity analysis, which examined weight status (adiposity) as a latent variable  *p<.05  **p<.01 | | | | | | |
