# Supplementary material for: The Influence of Depression, Positive Health Behaviors, and Weight Status on Glycated Hemoglobin: A Sequential Mediation Analysis of the INDEPENDENT Trial
Source: J Gen Intern Med. 2025 Aug 13;40(15):3715–22. doi: 10.1007/s11606-025-09810-1 (PMC12612419; doi:10.1007/s11606-025-09810-1)
Supplement: Supplementary file 5 — Supplementary file5 (PDF 118 KB) [file 11606_2025_9810_MOESM5_ESM.pdf]

Supplemental File 5- Path diagram visualizing the complex model which included intervention effects, cross-sectional associations between variables, mediation effects, and cross-sectional regression effects.

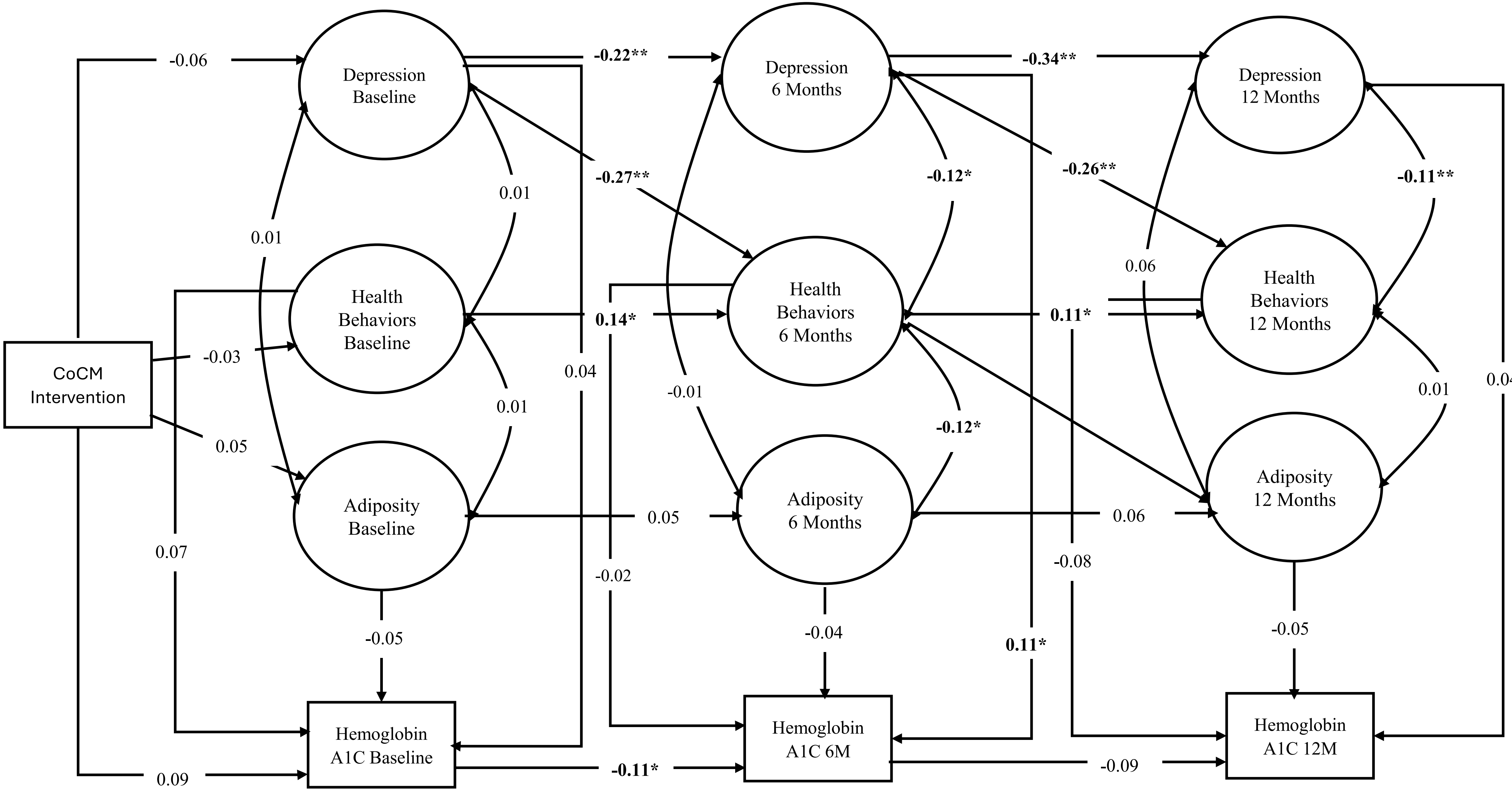

*p<.05, **p<.01 -Arrows drawn from baseline to 6-month scores and then 12 months represent the intervention effect at each wave on depression, health behaviors, and adiposity rather than autoregressive paths. - Circles represent latent variables - Boxes represent manifest variables - Single-headed arrows are causal pathways -Double-headed arrows are covariances
