# Supplementary material for: The Influence of Depression, Positive Health Behaviors, and Weight Status on Glycated Hemoglobin: A Sequential Mediation Analysis of the INDEPENDENT Trial
Source: J Gen Intern Med. 2025 Aug 13;40(15):3715–22. doi: 10.1007/s11606-025-09810-1 (PMC12612419; doi:10.1007/s11606-025-09810-1)
Supplement: Supplementary file 6 — Supplementary file6 (DOCX 28 KB) [file 11606_2025_9810_MOESM6_ESM.docx]

**Supplemental File 6: Results from Complex Structural Model**

**Complex Analysis 1: BMI**

| **Model** | **Path** | **Depression** | **Anhedonia** | **Restless** | **Somatic** | **Int. Dep** |
| --- | --- | --- | --- | --- | --- | --- |
| **Intervention Paths** | | | | | | |
| Treatment condition predicts depressive symptoms (a path) | a1 | -0.02 | 0.03 | -0.03 | -0.04 | 0.02 |
|  | a2 | **-0.20**** | **-0.11*** | 0.04 | -0.04 | 0.02 |
|  | a3 | **-0.35**** | **-0.23**** | -0.01 | 0.01 | 0.01 |
| Treatment condition predicts positive health behaviors (b path) | b1 | 0.02 | 0.02 | 0.02 | 0.02 | 0.02 |
|  | b2 | 0.06 | **0.15*** | **0.15*** | **0.15*** | **0.15*** |
|  | b3 | **0.11*** | 0.10 | 0.10 | 0.10 | 0.10 |
| Treatment condition predicts BMI (d path) | d1 | -0.01 | 0.01 | 0.01 | 0.01 | 0.01 |
|  | d2 | 0.01 | 0.01 | 0.01 | 0.01 | 0.01 |
|  | d3 | 0.02 | 0.02 | 0.02 | 0.02 | 0.02 |
| Treatment condition predicts A1C (c’ path) | c’1 | 0.05 | 0.09 | 0.08 | 0.08 | 0.08 |
|  | c’2 | -0.05 | **-0.11*** | **-0.12*** | **-0.12*** | **-0.12*** |
|  | c’3 | 0.03 | -0.08 | -0.09 | -0.10 | -0.10 |
| **Concurrent Paths** | | | | | | |
| Depression predicts A1C (e path) | e1 | 0.01 | 0.07 | 0.03 | 0.04 | 0.02 |
|  | e2 | **0.10*** | **0.12*** | 0.12 | 0.08 | 0.06 |
|  | e3 | 0.06 | 0.05 | 0.08 | 0.02 | 0.09 |
| Health Behaviors predict A1C (f path) | f1 | 0.08 | 0.11 | 0.10 | 0.10 | 0.10 |
|  | f2 | **-0.12*** | -0.02 | -0.02 | -0.04 | -0.06 |
|  | f3 | -0.06 | 0.02 | 0.05 | 0.01 | 0.04 |
| BMI Predicts A1C (g path) | g1 | 0.01 | -0.01 | -0.01 | -0.01 | -0.01 |
|  | g2 | 0.01 | -0.01 | 0.01 | -0.01 | 0.01 |
|  | g3 | 0.02 | 0.02 | 0.02 | 0.02 | 0.02 |
| **Cross-Lagged Paths** | | | | | | |
| Depression mediates Health behaviors (h path) | h1 | **-0.43**** | -0.02 | **-0.13*** | **-0.19*** | -0.01 |
|  | h2 | **-0.42**** | 0.01 | -0.01 | -0.01 | -0.03 |
| Health Behaviors mediate BMI (i path) | i1 | **-0.25*** | **-0.26*** | **-0.27*** | **-0.10*** | **-0.26*** |
|  | i2 | **-0.10*** |  |  |  |  |
| **-** A results table summarizing the effect sizes for the complex model, which included intervention effects, cross-sectional associations between variables, mediation effects, and cross-sectional regression effects.  *p<.05  **p<.01 | | | | | | |

**Complex Analysis 2: Waist Circumference**

| **Model** | **Path** | **Depression** | **Anhedonia** | **Restless** | **Somatic** | **Int. Dep** |
| --- | --- | --- | --- | --- | --- | --- |
| **Intervention Paths** | | | | | | |
| Treatment condition predicts depressive symptoms (a path) | a1 | -0.06 | 0.03 | -0.04 | -0.04 | 0.03 |
|  | a2 | **-0.23**** | **-0.11*** | 0.04 | -0.04 | 0.02 |
|  | a3 | **-0.34**** | **-0.23**** | -0.01 | 0.01 | 0.01 |
| Treatment condition predicts positive health behaviors (b path) | b1 | 0.02 | 0.02 | 0.02 | 0.02 | 0.02 |
|  | b2 | **0.15*** | **0.15*** | **0.15*** | **0.15*** | **0.15*** |
|  | b3 | 0.10 | 0.10 | 0.10 | 0.10 | 0.10 |
| Treatment condition predicts WC (d path) | d1 | 0.01 | 0.03 | 0.01 | 0.03 | 0.03 |
|  | d2 | 0.01 | -0.03 | 0.01 | -0.02 | -0.03 |
|  | d3 | 0.02 | -0.02 | 0.02 | -0.02 | -0.02 |
| Treatment condition predicts A1C (c’ path) | c’1 | 0.01 | 0.09 | 0.08 | 0.08 | 0.08 |
|  | c’2 | **-0.11*** | **-0.11*** | **-0.12*** | **-0.12*** | **-0.13*** |
|  | c’3 | -0.09 | -0.08 | -0.09 | -0.10 | -0.10 |
| **Concurrent Paths** | | | | | | |
| Depression predicts A1C (e path) | e1 | 0.06 | 0.07 | 0.03 | 0.04 | 0.03 |
|  | e2 | **0.12*** | **0.13*** | 0.12 | 0.08 | 0.06 |
|  | e3 | 0.07 | 0.05 | 0.09 | 0.02 | 0.09 |
| Health Behaviors predict A1C (f path) | f1 | 0.10 | 0.11 | 0.10 | 0.11 | 0.10 |
|  | f2 | -0.02 | -0.02 | -0.02 | -0.04 | -0.06 |
|  | f3 | 0.04 | 0.02 | 0.05 | 0.02 | 0.04 |
| WC Predicts A1C (g path) | g1 | -0.01 | -0.07 | -0.07 | -0.07 | -0.07 |
|  | g2 | -0.01 | -0.03 | -0.02 | -0.02 | -0.02 |
|  | g3 | 0.02 | -0.01 | -0.01 | -0.01 | -0.01 |
| **Cross-Lagged Paths** | | | | | | |
| Depression mediates Health behaviors (h path) | h1 | **-0.23**** | -0.01 | -0.10 | **-0.19*** | -0.02 |
|  | h2 | **-0.22**** | -0.01 | -0.01 | -0.01 | -0.01 |
| Health Behaviors mediate WC (i path) | i1 | **-0.20*** | -0.01 | -0.01 | -0.02 | -0.01 |
| *p<.05  **p<.01 | | | | | | |
